# A Simulation Model for Coupled Heat Transfer and Moisture Transport under the Action of Heat Source in Unsaturated Soils

**DOI:** 10.1038/s41598-018-26108-x

**Published:** 2018-05-17

**Authors:** Hua Jin, Yi Guo, Hongkai Deng, Xin Qi, Jinpeng Gui

**Affiliations:** 1 College of Water Resources Science and Engineering, Taiyuan University of Technology, Taiyuan, 030024 China; 2 Taiyuan Bilan Hydraulic Engineering Design Co. Ltd, Taiyuan, 030024 China; 3 The Melbourne School of Engineering, The University of Melbourne, Melbourne, Australia

## Abstract

Ground source heat pump (GSHP) system has been installed as the air-conditioning system worldwide due to it has the characteristics of high efficiency, easy access and environmental protection. Since ground heat exchanger (GHE) plays a key role in the performance of GSHPs, many models of GHE have been proposed to simulate temperature distribution around the borehole. However, most of these models depict only the heat conduction process between buried pipes and surrounding soil based on the line source model or cylindrical source model. And these models do not consider water transfer under the action of heat source, which can cause some prediction errors. The objective of this study is to provide a numerical model to simulate the spatiotemporal distribution of temperature and moisture caused by a GHE with constant temperature in unsaturated soils. The numerical model is developed by establishing two tridiagonal matrices and adopting Thomas algorithm to achieve the programming. The experiment is operated at the Taiyuan University of Technology and the comparisons between modeled and experimental data prove the high accuracy of this model. The model shows significant engineering values in designs and operations of GSHP.

## Introduction

Due to the shortage of fossil energy and the global warming becoming increasingly serious, improving energy consumption structure and reducing greenhouse gas emissions have become an urgent problem how to use a renewable energy as a substitute of the fossil energy. Compared with other renewable energy sources such as solar energy, wind energy, tidal energy, and biomass energy, shallow geothermal energy has the advantages of convenient access, stability and high efficiency. Hence, GSHP as a technology that utilizes shallow geothermal energy for space heating and cooling of residential, commercial and institutional buildings or hot water generating is widely used in the world as well as its high coefficient of performance (COP)^1–5^. The 80% of the energy demand in houses and buildings is currently used for the space heating and hot water producing^6^. Recently, the GSHPs installations have rapidly increased in the worldwide, especially in North American, Europe and China, and the number of countries that installed ground source heat pumps rose from 26 in 2000 to 48 in 2015^7^.

With the application and development of GSHPs, the model of GHE has been investigated by many researchers using analytical or numerical techniques validated with experimental measurements, because GHE plays a key role in configuration and performance of GSHPs. Most of these models depict the heat conduction process between buried pipes and surrounding soil based on the line source model or cylindrical source model. Chiasson *et al*.^8^ had made a preliminary investigation of the effects of groundwater flow on the heat transfer of vertical borehole heat exchangers. Diao. *et al*.^9^ had obtained an analytical solution on the heat transfer under coupled heat conduction and groundwater heat advection. Abdelaziz *et al*.^10^ developed a finite linesource model for vertical heat exchangers considering a layered soil profile. Conti *et al*.^11^ presented the numerical solution of the problem of an infinite cylindrical heat source embedded into a saturated porous medium. However, the above models are usually used to study heat transfer in saturated soil. A high-precision model of the GHEs in unsaturated soils is required for the purpose of optimal system design and operational control^12,13^.

Since horizontal GHEs are mainly installed in unsaturated soils, the research of the temperature variation in it shows the significant study value for GSHP designs and operations. Some investigators have conducted related studies on heat and moisture coupled transfer in unsaturated soil. Philip and de Vries^14^ first put forward a dual driving model considering temperature gradient and moisture gradient. Luikov^15^ developed a mathematical model, which takes into account the effects of the temperature gradient on the moisture migration. Li *et al*.^16^ presented an inner heat source model of underground heat exchanger based on the heat and mass transfer theory in the soil. Chen *et al*.^17^ presented an experimental study on the heat and moisture transfer in the soil for investigating heat charging and the coupled effect of heat and moisture transfer by one-dimensional soil column. Erdongan^18^ reported the moisture content in soil affected the size of ground heat exchangers by changing thermal properties of soil. Piechowski^19^ proposed a new approach which can result in a better accuracy value to simulate a horizontal type GHE, because of the consideration of heat and moisture transfer in the soil.

A number of researchers have carried out many valuable researches on heat and moisture transfer in unsaturated soil, but in the background of the GHEs, the studies on the coupled heat and moisture transfer in GHEs surrounding unsaturated soil are still limited. Therefore, it is necessary to strengthen the cooperation between the experiment and the model, and continue to study the high precision heat and moisture transfer models.

This paper firstly will review the previous studies on heat transfer progress and introduce several prediction models. Secondly, the new prediction model based on establishing two tridiagonal matrices will be explained. The paper will also describe the experiment and data collection and analyze the comparisons between modeled and experimental data. Finally, further improvements will also be discussed.

## Modeling

### Previous study

A number of mathematic models of GHEs for the heat transfer progress have appeared since the first model called Kelvin’s line source was developed^20^. Yang^21^ review the history of ground heat transfer study and divide these models into three types based on different methods: analytical methodology, numerical method and the combination of both of them. However, there are limited studies on the model for the coupling progress of heat and moisture transfer caused by the GSHP operation.

Since the moisture transfer also carries some heat, the ground heat and moisture transfer are actually coupled^14^. Thomas^22^ explains the mathematic analysis of the heat and moisture transfer in unsaturated soils. Abu-Hamdeh and Reeder^23^ also state that the rising moisture content can increase the ground thermal conductivity. This paper, therefore, introduces a tridiagonal matrix to simulate the spatiotemporal distribution of the heat and moisture transfer caused by the heat source with constant temperature in unsaturated soils.

### Simplifying assumption

Following are the basic assumptions of this model:

1. The soil is homogeneous;
2. The moisture content consists of pure water;
3. Gravity is neglected;
4. GHE has a constant temperature;
5. The influence of air is neglected due to its limited impacts^24^;
6. Soil thermal conductivity and heat capacity are both constant.

Since soil is homogeneous, the coupling transfer of heat and moisture can be concluded that each direction of the progress is symmetrical by regarding the heat source as the center. As a result, the spatial distribution is simplified as a one-dimensional soil column model. Distance and time, therefore, will become the only two independent variables of this model.

### Initial and boundary conditions

Initial temperature and moisture both are uniform through the one-dimensional soil column. The temperature of the soil at zero distance is assumed to be the heat source temperature, due to the neglect of the heat transfer time. Since the soil column is assumed to have a long length, both the temperature and moisture at the outer of it will be constant and uninfluenced.

### Equations and physical meanings

As the heat and moisture transfers are coupled in the ground, this paper combines the mass conservation equation of water content and energy conservation equation to achieve the simulation objective. Followings are the differential equations:

The equation of moisture mass conservation explains the moisture transfer progress^25^:

1 $$\frac{\partial S}{\partial t}={D}_{s}\frac{{\partial }^{2}S}{\partial {x}^{2}}+{D}_{T}\frac{{\partial }^{2}T}{\partial {x}^{2}}$$

where *S* is the moisture content; *T* is the temperature; *t* is the time; *x* is the distance; *D*_*S*_ is mass diffusivity under the moisture content gradient; *D*_*T*_ is the mass diffusivity under the temperature gradient.

The equation of energy conservation explains the heat transfer progress^14^:

2 $$\rho c\frac{\partial T}{\partial t}=\frac{\partial }{\partial x}(\lambda \frac{\partial T}{\partial x})+{D}_{s}\varphi {\rho }_{l}{c}_{l}\frac{\partial S}{\partial x}\frac{\partial T}{\partial x}$$

where *ρ* and *ρ*_*l*_ are the soil and moisture density respectively; *λ* is the thermal conductivity of soil; *c* and *c*_*l*_ are the heat capacities of soil and moisture respectively; *ϕ* is the soil porosity.

### Discretization and matrix derivation

The adopted discretization used in this experiment is the finite difference method (FDM), which is similar to the numerical method produced by Li and Zheng^26^. Time and distance are discretized into a finite number of time steps and contiguous column elements respectively.

As a result, Eqs (1) is derived into a tridiagonal matrix:

3 $$\begin{array}{c}{[\begin{array}{cccccc}1+2{a}_{1} & -{a}_{1} & 0 & \cdots  & \cdots  & 0\\ -{a}_{1} & 1+2{a}_{1} & -{a}_{1} & \vdots  & \vdots  & \vdots \\ 0 & 0 & \ddots  & 0 & 0 & 0\\ \vdots  & \cdots  & 0 & -{a}_{1} & 1+2{a}_{1} & -{a}_{1}\\ 0 & \cdots  & \cdots  & 0 & -{a}_{1} & 1+2{a}_{1}\end{array}]}_{(M-1)\times (M-1)}\times {[\begin{array}{c}\begin{array}{c}{S}_{1}^{n}\\ {S}_{2}^{n}\end{array}\\ \vdots \\ \begin{array}{c}{S}_{M-2}^{n}\\ {S}_{M-1}^{n}\end{array}\end{array}]}_{(M-1)\times 1}\\ =\,{[\begin{array}{c}\begin{array}{c}{S}_{1}^{n-1}+{a}_{1}{S}_{0}^{n}\\ {S}_{2}^{n-1}\end{array}\\ \vdots \\ \begin{array}{c}{S}_{M-2}^{n-1}\\ {S}_{M-1}^{n-1}+{a}_{1}{S}_{M}^{n}\end{array}\end{array}]}_{(M-1)\times 1}+{b}_{1}{[\begin{array}{c}\begin{array}{c}{T}_{2}^{n}-2{T}_{1}^{n}+{T}_{0}^{n}\\ {T}_{3}^{n}-2{T}_{2}^{n}+{T}_{1}^{n}\end{array}\\ \vdots \\ \begin{array}{c}{T}_{M-1}^{n}-2{T}_{M-2}^{n}+{T}_{M-3}^{n}\\ {T}_{M}^{n}-2{T}_{M-1}^{n}+{T}_{M-2}^{n}\end{array}\end{array}]}_{(M-1)\times 1}\end{array}$$

4 $${a}_{1}=\frac{{D}_{s}{\rm{\Delta }}t}{{({\rm{\Delta }}x)}^{2}}$$

5 $${b}_{1}=\frac{{D}_{T}{\rm{\Delta }}t}{{({\rm{\Delta }}x)}^{2}}$$

where *M* is the total number of column elements; *n* is the total number of time steps.

The matrix model for energy conservation equation is:

6 $$[\begin{array}{cccccc}{c}_{1} & -{a}_{2} & 0 & \cdots  & \cdots  & 0\\ {d}_{2} & {c}_{2} & -{a}_{2} & \vdots  & \vdots  & \vdots \\ 0 & 0 & \ddots  & 0 & \,0\, & 0\\ \vdots  & \,\cdots \, & 0 & {d}_{M-2} & {c}_{M-2} & -{a}_{2}\\ 0 & \,\cdots \, & \cdots  & 0 & {d}_{M-1} & {c}_{M-1}\end{array}][\begin{array}{c}\begin{array}{c}{T}_{1}^{n}\\ {T}_{2}^{n}\end{array}\\ \vdots \\ \begin{array}{c}{T}_{M-2}^{n}\\ {T}_{M-1}^{n}\end{array}\end{array}]=[\begin{array}{c}\begin{array}{c}{T}_{1}^{n-1}-{d}_{1}{T}_{0}^{n}\\ {T}_{2}^{n-1}\end{array}\\ \vdots \\ \begin{array}{c}{T}_{M-2}^{n-1}\\ {T}_{M-1}^{n-1}+{a}_{2}{T}_{M}^{n}\end{array}\end{array}]$$

7 $${a}_{2}=\frac{\lambda {\rm{\Delta }}t}{\rho c{({\rm{\Delta }}x)}^{2}}$$

8 $${b}_{2}=\frac{{D}_{s}\varphi {\rho }_{l}{c}_{l}{\rm{\Delta }}t}{\rho c{({\rm{\Delta }}x)}^{2}}$$

9 $${c}_{i}=(1+2{a}_{2})-{b}_{2}({S}_{i}^{n}-{S}_{i-1}^{n})$$

10 $${d}_{i}={b}_{2}({S}_{i}^{n}-{S}_{i-1}^{n})-{a}_{2}$$

For an element linear equations *Ax* = *d*:

11 $$[\begin{array}{ccccc}{e}_{1} & {f}_{1} &  &  & \\ {g}_{1} & {e}_{2} & {f}_{2} &  & \\  & \ddots  & \ddots  & \ddots  & \\  &  & \ddots  & \ddots  & {f}_{n-1}\\  &  &  & {g}_{n-1} & {e}_{n}\end{array}][\begin{array}{c}\begin{array}{c}{x}_{1}\\ {x}_{2}\end{array}\\ \vdots \\ \begin{array}{c}{x}_{n-1}\\ {x}_{n}\end{array}\end{array}]=[\begin{array}{c}\begin{array}{c}{d}_{1}\\ {d}_{2}\end{array}\\ \vdots \\ \begin{array}{c}{d}_{n-1}\\ {d}_{n}\end{array}\end{array}]$$

If the matrix *A* meets the following conditions:

12 $$\{\begin{array}{c}|{e}_{1}| > |{f}_{1}|\\ |{e}_{i}| > |{f}_{i}|+|{g}_{i-1}|;(i=2,3,\cdots ,n-1)\,\\ |{e}_{n}| > |{g}_{n-1}|\\ {e}_{i}\ne 0\,;\,(i=1,2,\cdots ,n)\end{array}$$

The result can be solved by using Thomas algorithm^27^.

Put in details, for the matrix *A*:

13 $$A=\tilde{L}\tilde{U}=[\begin{array}{ccccc}{p}_{1} &  &  &  & \\ {r}_{1} & {p}_{2} &  &  & \\  & \ddots  & \ddots  &  & \\  &  & \ddots  & \ddots  & \\  &  &  & {r}_{n-1} & {p}_{n}\end{array}]\,[\begin{array}{ccccc}1 & {q}_{1} &  &  & \\  & 1 & {q}_{2} &  & \\  &  & \ddots  & \ddots  & \\  &  &  & \ddots  & {q}_{n-1}\\  &  &  &  & 1\end{array}]$$

Followings are the specific decomposition formulas for solving $\tilde{L}y=d$ and $\tilde{U}x=y$:

14 $$\{\begin{array}{c}{p}_{1}={e}_{1}\\ {r}_{i}={g}_{i}\\ {q}_{i}=\frac{{f}_{i}}{{p}_{i}}\\ {p}_{i+1}={e}_{i+1}-{r}_{i}{q}_{i}\end{array}(i=1,2,3\,\cdots n-1)$$

15 $$\{\begin{array}{c}{y}_{1}=\frac{{d}_{1}}{{p}_{1}}\\ {y}_{i}=({d}_{i}-{r}_{i-1}\,{y}_{i-1})/{p}_{i};(i=2,3\,\cdots n)\\ {x}_{n}={y}_{n}\\ {x}_{i}={y}_{i}-{q}_{i}{x}_{i+1}\,;(i=n-1,n-2,\cdots ,1)\end{array}$$

After solving $\tilde{L}y=b$ and $\tilde{U}x=y$, the result of the matrix *A* is derived.

For the matrices of this study, since:

16 $$\{\begin{array}{c}|1+2{a}_{1}| > |-{a}_{1}|\\ |1+2{a}_{1}| > |-{a}_{1}|+|-{a}_{1}|\\ |1+2{a}_{1}|\ne 0\end{array}$$

And

17 $$\{\begin{array}{c}|(1+2{a}_{2})-{b}_{2}({S}_{1}^{n}-{S}_{0}^{n})| > |-{a}_{2}|\\ |(1+2{a}_{2})-{b}_{2}({S}_{i}^{n}-{S}_{i-1}^{n})| > |-{a}_{2}|+|{b}_{2}({S}_{i}^{n}-{S}_{i-1}^{n})-{a}_{2}|;\\ (i=2,3,\cdots ,M-2)\\ \begin{array}{c}|(1+2{a}_{2})-{b}_{2}({S}_{M-1}^{n}-{S}_{M-2}^{n})| > |{b}_{2}({S}_{M-1}^{n}-{S}_{M-2}^{n})-{a}_{2}|\\ |(1+2{a}_{2})-{b}_{2}({S}_{i}^{n}-{S}_{i-1}^{n})|\ne 0;(i=1,2,\cdots ,M-1)\end{array}\end{array}$$

Thomas algorithm can be adopted in solving both equations. As a result, the simulation model for predicting the specific spatiotemporal distribution of temperature and moisture is developed by combining these two matrices. For the example study, this paper uses MATLAB to achieve the programming.

## Experimental Study

### Equipment description

Since the study focuses on the model of a one-dimensional soil column. The adopted equipment is similar to the experiment described by Chen *et al*.^28^. And the whole experiment was operated at the Taiyuan University of Technology.

**Figure 1:**
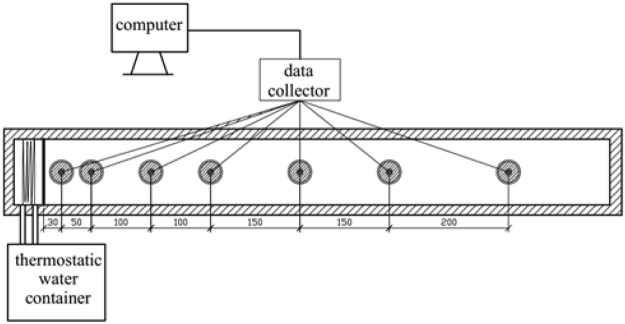
Experimental equipment (unit: mm).

**Figure 2:**
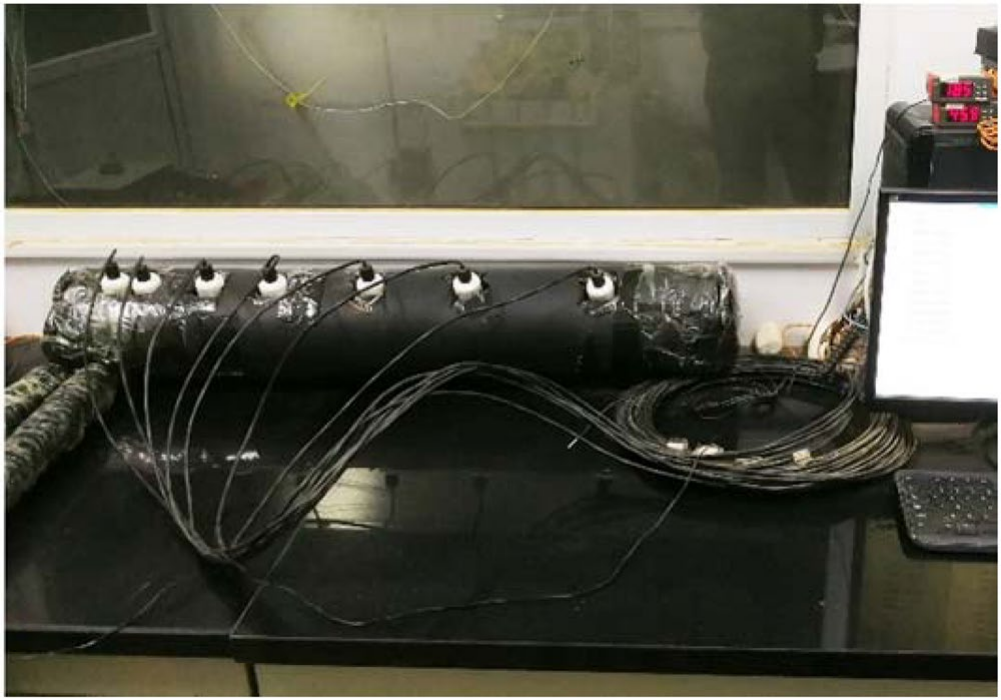
Actual picture of the equipment.

Figure 1 shows the equipment diagram and Fig. 2 is the picture of actual experimental equipment. The equipment consists of a soil column, Stevens Hydra Probe II Soil Moisture Sensors, a data collector (Campbell CR200), a thermostatic water container (BILON HX-80), NTC temperature sensors, a compaction hammer, insulation devices, a transformer, and a computer.

### Procedure description

The water container provides the heat for the heat source at the left side of the soil column to keep it at a constant temperature. And, the temperature of the heat source can be detected by the NTC sensor. The soil column has seven observation points. For each point, the soil moisture sensor can detect the soil temperature and moisture at the same time^29^. Table 1 shows the parameters of this sensor’s accuracy and precision. Additionally, all the data can be collected and organized in the computer. The operating period of each experiment is 24 hours.

**Table 1:** Accuracy and precision of Stevens Hydra Probe II Soil Moisture Sensor.

Parameters	Accuracy/Precision
Temperature (°C)	+/− 0.6 Degrees Celsius (From −30 °C to 36 °C)
Soil Moisture wfv^†^ (m^3^∙m^−3^)	+/− 0.03 wfv (m^3^∙m^−3^) Accuracy (Typical)
Soil Moisture wfv^†^ (m^3^∙m^−3^)	+/− 0.003 wfv (m^3^∙m^−3^) Precision
Electrical Conductivity^†^ (S/m) TUC*	+/− 0.0014 S/m or +/− 1% (Typical)
Electrical Conductivity^†^ (S/m) TC**	+/− 0.0014 S/m or +/− 5% (Typical)
Real/Imaginary Dielectric Constant TUC*	+/− 0.5 or +/− 1%
Real/Imaginary Dielectric Constant TC*	+/− 0.5 or +/− 5%

*TUC Temperature uncorrected full scale.

**TC Temperature corrected from 0 to 35 °C.

^†^The Accuracy and precision of the soil moisture, and EC measurements as well as the temperature corrections, are highly soil dependent.

## Comparison between modeled and experimental data

The paper will introduce two experimental results as examples to verify the model’s accuracy. Two experiments use the same soil and the major difference is the temperature of the heat source. Table 2 shows the parameters of each comparison. The soil thermal conductivity and heat capacity are cited from Duan^30^.

**Table 2:** Related parameters of the comparison.

Parameters	Experiment 1	Experiment 2
Soil thermal conductivity (J/(min∙m∙°C))	48	48
Soil particle density (kg/m^3^)	2650	2650
Heat capacity of soil(J/(kg∙°C))	1739	1739
Length of soil column (m)	1	1
Number of distance element	100	100
Time step (min)	1	1
Total time (h)	24	24
Initial soil temperature (°C)	20.6	19.3
Temperature of heat source (°C)	33.5	24.5
Porosity	0.4403	0.4403
Water thermal conductivity (J/(min∙m∙°C))	35.94	35.94
Water density (kg/m^3^)	1000	1000
Heat capacity of moisture (J/(kg∙°C))	4180	4180
Initial moisture content (m^3^/m^3^)	0.08	0.08

### Heat transfer progress

**Figure 3:**
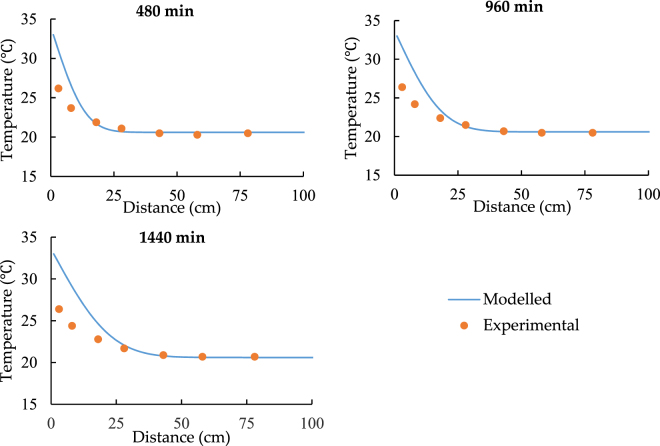
The spatial distribution of soil temperature with 33.5 °C heat source.

**Figure 4:**
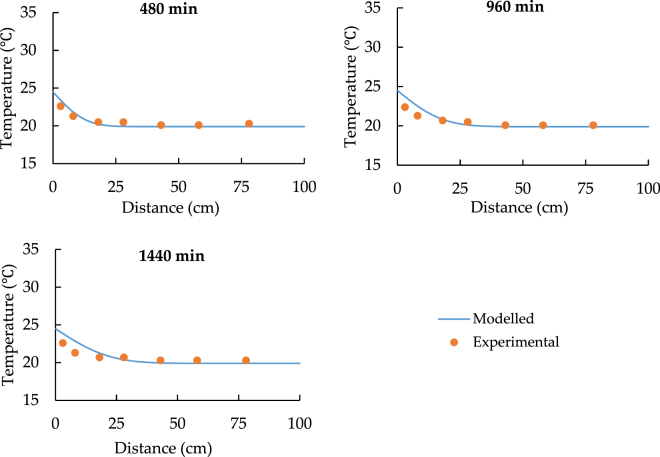
The spatial distribution of soil temperature with 24.5 °C heat source.

**Figure 5:**
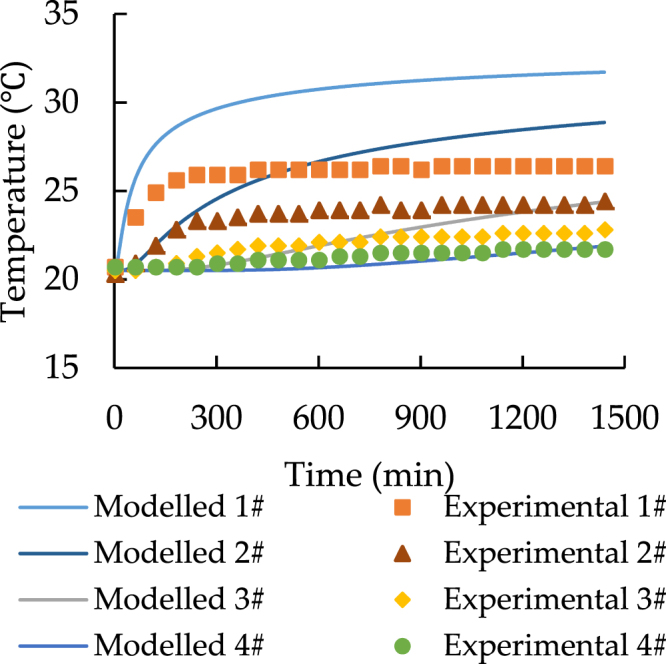
Temporal distribution of soil temperature with 33.5 °C heat source.

**Figure 6:**
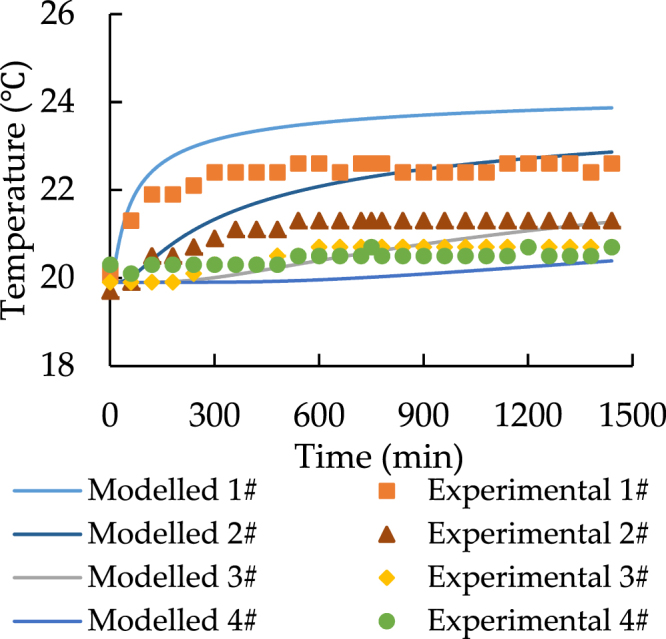
Temporal distribution of soil temperature with 24.5 °C heat source.

Figures 3 to 6 show the results of two different experiments under the different temperature of the heat source. All the charts indicate that the model can predict the similar trend as the experiments. According to Fig. 3, at the distance from 0 to 20 cm, model values are clearly larger than the experimental data and the maximum difference of temperature is around 5 °C. Figure 4 shows that the temperature of soil under 24.5 °C heat source is nearly constant. It also indicates that if the temperature of heat source and soil has the limited difference at the start of the transfer progress, the model shows much better performance and the relative error varies from −5% to 1.5%.

According to Figs 3 and 4, point 5 to 7 have a constant temperature during the transfer progress. As a result, the comparisons should be focused on the data for point 1 to 4. Both Figs 5 and 6 prove that the model can simulate the similar trend as the experiment. Although the model has higher performance at point 3 and point 4, the accuracy of its prediction is much lower at point 1 and 2.

In conclusion, the comparison of the spatiotemporal distribution of soil temperature illustrates that the model has low accuracy in predicting the soil temperature at the distance while the soil is closed to the heat source. Additionally, Figs 3–6 also indicate that at the shorter distance, the model data is always higher than the experimental data. As a result, the error of comparison is systematic. The main reasons are the thermal contact resistance between soil and the heat source wall, the heat losses during the experiment, and the heat source temperature up to planned temperature needs some time at the start of each experiment.

### New comparison after the adjustment

#### Modification of the Heat Source temperature

According to the aforementioned analysis, the factors that can influence the model precision are complex, it is difficult to quantify each factor in details. However, in order to improve the prediction accuracy of the model, the appropriate modified heat source temperature can be used instead of the initial heat source temperature in the model. The modified heat source temperature is obtained by fitting equation of temperature spatial distribution curve. According to Figs 3 and 4, we can see that the temperature spatial distribution curve is close to the exponential function distribution, thus the exponential function deformation formula is used to fit the curve. Following is the fitting equation:

18 $$T=a+b\cdot \exp (cx)$$

where *a, b, c* are constants; *T* is the temperature; x is the distance from heat source wall.

**Figure 7:**
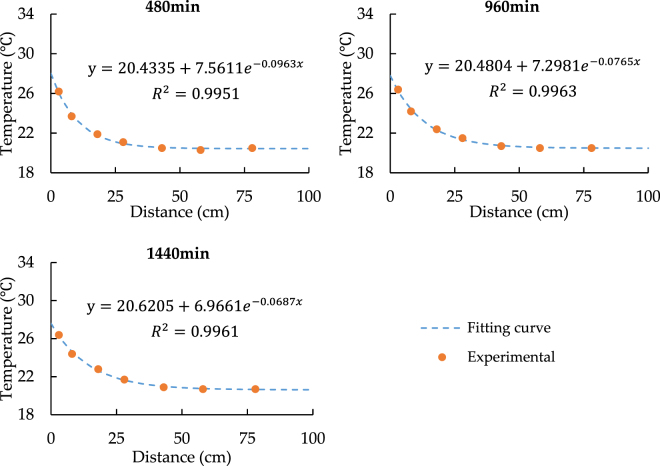
Fitting Curves of temperature Spatial Distribution with 33.5 °C.

**Figure 8:**
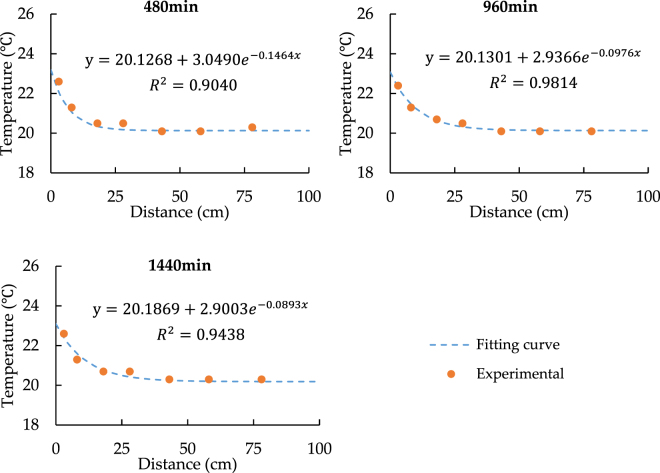
Fitting Curves of temperature Spatial Distribution with 24.5 °C heat source.

The fitting results of the temperature distribution curves with the heat source of 33.5 °C and 24.5 °C heat source are shown in Figs 7 and 8.

According to Figs 7 and 8, it can be seen that the *T* values are around 28 °C and 23 °C respectively while x = 0 cm. Therefore, we can regard 28 °C and 23 °C as the modified heat source temperatures and replace 33.5 °C and 24.5 °C in the model.

#### Comparison after the adjustment temperature of heat source

The temperature of the heat source is changed in the further model validation. The new heat source temperatures of experiment 1 and 2 are 28 °C and 23 °C separately. Following figures can illustrate the new comparisons after the adjustments.

**Figure 9:**
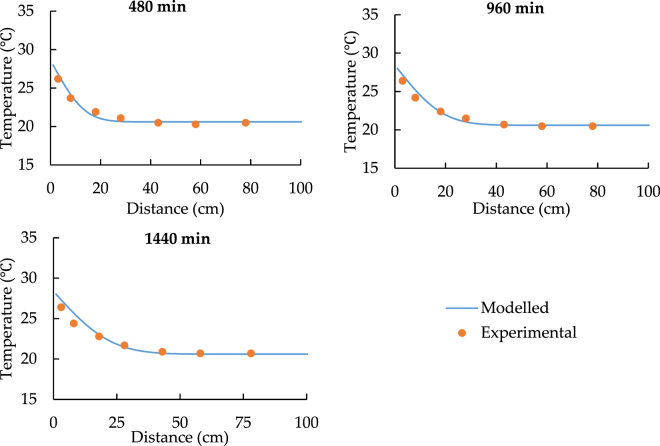
The spatial distribution of soil temperature with 28 °C heat source after the adjustment.

**Figure 10:**
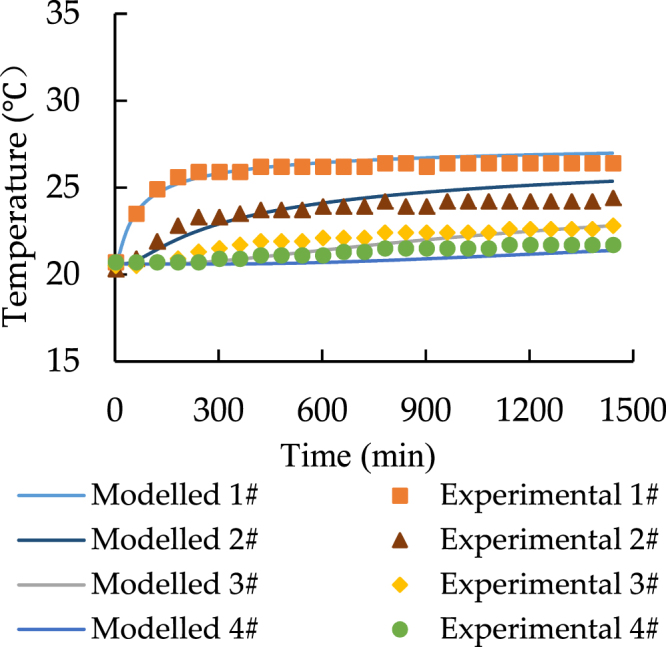
The temporal distribution of soil temperature with 28 °C heat source after the adjustment.

**Figure 11:**
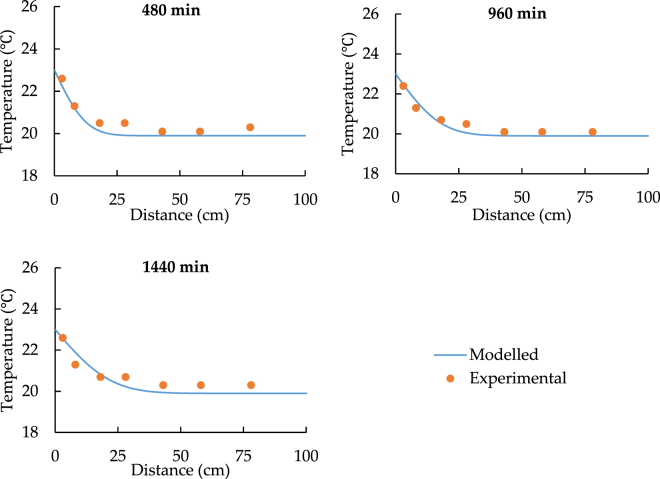
The spatial distribution of soil temperature with 23 °C heat source after the adjustment.

**Figure 12:**
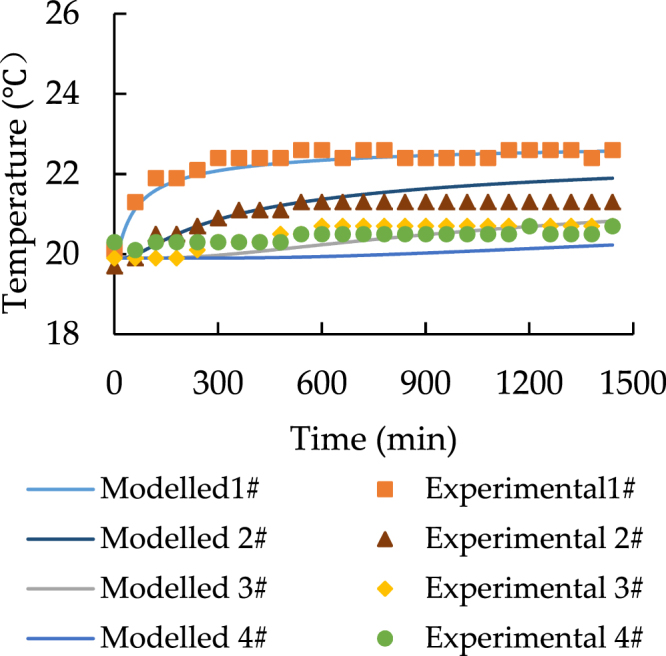
The temporal distribution of soil with 23 °C heat source after the adjustment.

Figures 9–12 show the new comparison after the adjustment of the heat source temperature and the maximum relative error is only around 3%. It can be concluded that both modeled and experimental data achieve a good agreement.

### Moisture transfer progress

According to the results of heat transfer comparison, the temperature of the point 5 to 7 can be concluded to be constant, and it will not become the major factor which influences the moisture content. Therefore, following figures for the spatial distribution will only discuss the results of point 1 to 4.

**Figure 13:**
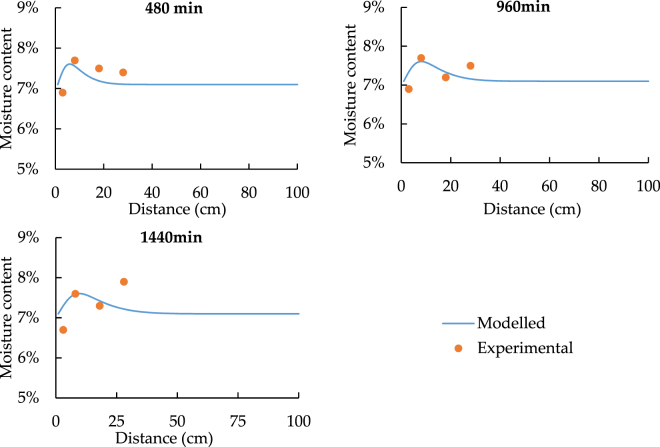
The spatial distribution of soil moisture with 33.5 °C heat source.

**Figure 14:**
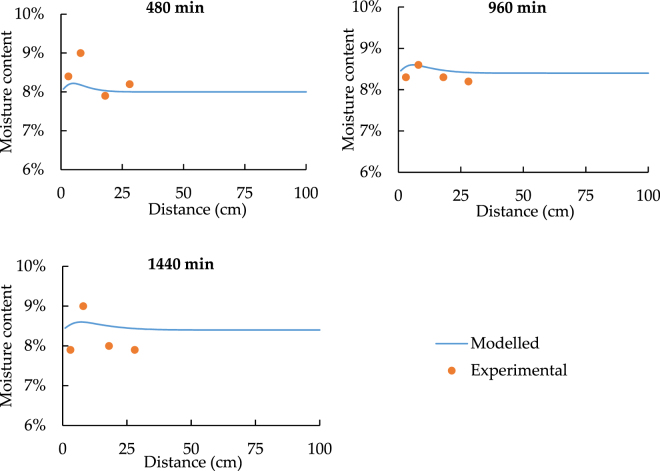
The spatial distribution of soil moisture with 24.5 °C heat source.

Since the sensors have 3% accuracy error of detecting moisture content, the value differences are all acceptable. As a result, in order to confirm the model performance, similar time changing trend has much higher importance. Besides this, Figs 13 and 14 state that the moisture content becomes nearly constant at point 3. Hence, for the temporal distribution, only point 1 and point 2 have the clear changing trend.

**Figure 15:**
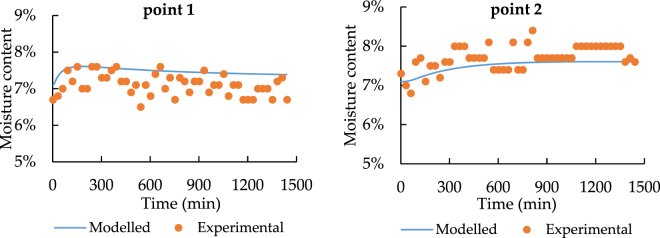
Temporal distribution of soil moisture with 33.5 °C heat source.

**Figure 16:**
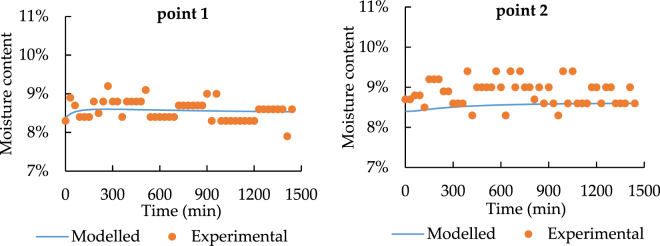
Temporal distribution of soil moisture with 24.5 °C heat source.

According to Figs 15 and 16, the modeled and experimental data achieve a good agreement on the trend with time changing at point 1 and 2, which means that the model has a high accuracy of predicting moisture transfer progress.

## Discussion

In this paper, the characteristics of heat and moisture migration in unsaturated soil were studied by means of experimental verification and numerical simulation. The results in Figs 3 and 7 or Figs 4 and 9 showed that the model of the modified heat source temperature (28 °C or 23 °C) gave more accurate prediction compared to the observed heat source temperature (33.5 °C or 24.5 °C). The higher the heat source temperature is, the higher the adjustment of the heat source temperature will be. Additionally, the larger difference between heat source temperature and soil temperature is, the greater the regulation temperature of the heat source will be. In the numerical simulation, because the thermal contact resistance between the wall of the heat source and the soil directly affects the characteristics of heat transfer, the heat source temperature was regulated in order to improve the simulated accuracy. As mentioned in section 4.1 there are three main reasons why can cause the system error of comparison, and the thermal contact resistance is the most important factor among those three. The thermal contact resistance causes the temperature drop at the interface between the surfaces in contact of materials, and is influenced by the type of the materials, the surface roughness, the interface temperature, and the heat flow direction, etc^31^. Piechowski concluded that the steepest temperature gradient in the soil region occurs at the pipe-soil interface, and the soil temperature reduced by up to 30–40% away from the pipe surface^19^. Furthermore, due to the gaps and spots of contact interface existed, only in some discrete areas, a very small proportion of the nominal contact area, actually contact perfectly^32^. There is a temperature drop and additional resistance has occurred at the interface. Therefore, the temperature values adjusted at the soil 0 cm in the simulation was obtained using the empirical formula by fitting seven measuring points in the experiment of the soil column, and the simulation results achieve a good agreement of both simulated and experimental data.

In addition, the moisture transfer trend under the action of different heat source temperature is similar. The higher the heat source temperature is, the larger amount of moisture migration will appear. This water migration process leads to a lower moisture content near the heat source wall^17^. Since the changes of moisture content can affect the thermal properties of unsaturated soil, the inhomogeneity of water content distribution means heat transfer process is complex in actual.

In order to improve the precision of the heat and moisture coupled transfer model, the following factors may be useful to consider. Firstly, the thermal conductivity of soil particles is not constant in the actual environment. It can be changed with temperature and moisture of the soil. Therefore, the soil thermal conductivity may increase or decrease during temperature and humidity changing. Another improvement is the consideration of the thermal contact resistance, and it still has significant effects on the heat transfer progress in the interface of different materials.

## Conclusion

Based on the theory of coupled heat and moisture transfer, the objective of this paper is developing a tridiagonal matrix for predicting this coupling progress under the action of the heat source with constant temperature in unsaturated soils. The development of this model is based on the equations of energy and moisture mass conservation. Comparisons of modeled and experimental figures are also adopted in verifying the simulation accuracy.

In addition, the accuracy of the model can be greatly improved by proper adjusting the heat source temperature by fitting the curve of soil temperature distribution considering the effect of the thermal contact resistance etc. And the results indicate that the model has significant values in GSHP projects.

Still, there are some limitations in this study, such as the varieties of soil thermal physical parameters are not considered and the thermal contact resistance between buried pipe and soil are not analyzed in details. Further researches can be carried out from solving the existing limitations.

### Data availability

The datasets generated during and/or analysed during the current study are available from the corresponding author on reasonable request.
